# A new species of *Solanum* (Solanaceae) from South Africa related to the cultivated eggplant

**DOI:** 10.3897/phytokeys.8.2462

**Published:** 2012-01-01

**Authors:** M.S. Vorontsova, S. Knapp

**Affiliations:** 1 Herbarium, Library and Archives, Royal Botanic Gardens, Kew, Richmond, Surrey TW9 1AD, United Kingdom; 2 Department of Botany, The Natural History Museum, Cromwell Road, London SW7 5BD, United Kingdom

**Keywords:** Africa, andromonoecy, eggplant, endemic, South Africa, “spiny solanum”

## Abstract

A new andromonoecious species related to the eggplant and belonging to *Solanum* subgenus *Leptostemonum* from southern Africa is described. *Solanum umtuma* Voronts. & S.Knapp, **sp. nov.** is found in the eastern part of South Africa, and is sympatric with its close relative *Solanum linnaeanum* Hepper & P.M-L.Jaeger. It is morphologically very similar to *Solanum cerasiferum* Dunal of northern tropical Africa. A comparison table with similar and closely related species is provided, as are a distribution map and illustration of *Solanum umtuma*.

## Introduction

The Solanaceae is an economically important, cosmopolitan family with approximately 3000 species in some 90 genera. The Solanaceae include globally important food crops such as the cultivated potato (*Solanum tuberosum* L.), tomato (*Solanum lycopersicum* L.), aubergine (*Solanum melongena* L.), and chilli pepper (*Capsicum* spp.) as well as a number of widely used drug plants such as tobacco (*Nicotiana tabacum* L.), *Datura*, and *Atropa belladonna* L., the source of atropine. The giant genus *Solanum* L. with ca. 1500 species has become a model system for collaborative online taxonomy in challenging tropical plant groups (see Knapp et al. 2004 and http://www.solanaceaesource.org). Many new species of *Solanum* have been described as part of the PBI (Planetary Biodiversity Inventory) *Solanum* project (e.g. Knapp 2010; Vorontsova et al. 2010a, 2010b; Vorontsova and Mbago 2011) that aims to produce a complete species-level on-line monograph of the genus. In the course of work on the prickly solanums in Africa and Madagascar we discovered the new species described here.

The “spiny” (or more accurately prickly) solanums (*Solanum* subgenus *Leptostemonum* Dunal) are the largest clade in the genus, with some 750 species (Whalen 1984; Bohs 2005). Most species in subgenus *Leptostemonum* are found in the New World, but approximately 150 species occur in the Old World, including taxa from Africa, Asia, and Australia. These Old World species form a monophyletic clade (see Levin et al. 2006; Weese and Bohs 2010). In the Old World clade of prickly solanums, the wild relatives of the cultivated eggplant (or aubergine) *Solanum melongena* are one of the most variable and confusing groups. They have been classified as *Solanum* section *Melongena* (Mill.) Dunal (Bitter 1923) or the *Solanum incanum* species group (Whalen 1984), and many taxa, both at the specific and infraspecific rank, have been described for these variable plants.

Although the eggplant is generally considered to be a vegetable of Asian origin and distribution (see Wang et al. 2008), it is a member of a predominantly African clade within the prickly solanums (Weese and Bohs 2010). In addition to *Solanum melongena* (including *Solanum ovigerum* Dunal) the “wild eggplants” currently include seven species of prickly subshrubs native to Africa and Asia: *Solanum aureitomentosum* Bitter, *Solanum campylacanthum* Hochst. ex A.Rich. (including *Solanum panduriforme* Dunal and *Solanum delagoense* Dunal), *Solanum cerasiferum* Dunal, *Solanum incanum* L., *Solanum insanum* L. (including *Solanum cumingii* Dunal), *Solanum lichtensteinii* Willd., and *Solanum linnaeanum* Hepper & P.-M.L.Jaeger (for complete synonymy of these taxa see the Solanaceae Source website, http://www.solanaceaesource.org; complete synonymy will also be included in the upcoming monograph). These species are all bushy erect subshrubs 0.5–2 m tall with lobed leaves, an andromonoecious breeding system with 1(-3) larger hermaphrodite flowers at the base of every inflorescence and smaller functionally male flowers at the distal parts of inflorescences, 1(-3) large yellow fruits, and variable pubescence composed of stellate trichomes. They occupy similar ecological niches throughout their respective ranges (see Table 1 for a comparison of the accepted species in this group) and usually are found growing in open disturbed areas between sea level and approximately 2000 m elevation. Complex species boundaries and high levels of morphological variability have led to much confusion between these species of eggplant relatives, and all of them have been placed in *Solanum incanum* sensu lato at one time or another (e.g., in floristic works such as D’Arcy and Rakotozafy 1994; Gonçalves 2005), with the exception of the morphologically quite distinct *Solanum linnaeanum* with rounded leaf lobes that has historically been called “*Solanum sodomeum* L.” (Hepper and Jaeger 1986). *Solanum linnaeanum* is probably native to southern Africa although it is a common weed in North Africa and southern Europe.

**Table 1.** Morphological differences between *Solanum umtuma* and other eggplant relatives in Africa. Calyx characters refer to the long-styled flowers at the base of the inflorescence and not to the smaller, functionally male, short-styled flowers in the distal parts of the inflorescence. This comparison focuses on the characters relevant to the identification of *Solanum umtuma* and does not include all the characters useful for separating the other members of this group (these will be presented in an up-coming monographic treatment of the prickly solanums of Africa and Madagascar, Vorontsova and Knapp in prep.).

	**Leaf shape**	**Leaf base**	**Apices of primary leaf lobes**	**Secondary leaf lobes**	**Total calyx length**	**Calyx lobe length**	**Calyx lobe shape**	**Calyx lobe apex**	**Prickle # on calyx at anthesis**	**Distribution**
*Solanum aureitomentosum*	ovate	obtuse to cordate	rounded to obtuse	absent	12–19 mm	7–10 mm	ovate to oblong and foliaceous	obtuse	30–60	Southern Africa, from southern DR Congo to Angola, southern Tanzania, Zambia, and Zimbabwe
*Solanum campylacanthum*	ovate to elliptic or lanceolate	rounded to cordate	rounded, sometimes acute	absent	7–15 mm	5–10 mm	deltate to narrow-deltate	acute to obtuse or acuminate	0–20	Ubiquitous weed of low altitudes in Southern and Eastern Africa
*Solanum cerasiferum*	ovate to elliptic	attenuate	rounded to acute	sometimes present	7–12 mm	4–7 mm	deltate to narrow-deltate	acuminate	0–20	From Senegal to Cameroon, Sudan and Ethiopia
*Solanum incanum*	ovate	rounded to cordate	rounded	absent	6–10 mm	2.5–5 mm	deltate to narrow-deltate	acute to obtuse	15–60	Predominantly in Ethiopia, Somalia, Arabia, and India, with some populations in N Kenya, Sudan, and extending to Mali
*Solanum insanum*	ovate	truncate, sometimes obtuse	rounded	absent	5–10 mm	4–6 mm	deltate	acute	0–15	Madagascar, India to SE Asia
***Solanum umtuma***	**elliptic**	**cuneate to truncate**	**obtuse to acute**	**often present**	**11–22 mm**	**7–10 mm**	**ovate and foliaceous**	**bluntly acute**	**30–80**	**South Africa**
*Solanum lichtensteinii*	ovate	cordate, sometimes cuneate	rounded	absent	7–15 mm	3.5–6 mm	deltate to narrow-deltate	acute to obtuse	20–50	Angola to South Africa, DR Congo, and Tanzania
*Solanum linnaeanum*	elliptic, sometimes ovate or obovate	cuneate or obtuse	rounded	always present and often well-developed	10–14 mm	5–6 mm	deltate to ovate	acute to rounded	30–100	Native to South Africa and naturalised in disturbed, often coastal, habitats worldwide
*Solanum melongena*	ovate	cordate to obtuse	rounded	absent	10–40 mm	5–17 mm	deltate to narrow-deltate	acute to long-acuminate	0(-30)	Cultivated worldwide (commonly cultivated in West Africa, sometimes in southern Africa, rarely cultivated in tropical Africa)

Work on species limits in the eggplant group was carried out by the late Richard Lester’s students (Jaeger 1985; Hasan 1989; Lester and Hasan 1991; Samuels 1994, 1996) using morphological and biosystematic methods. Molecular phylogenetic reconstruction by Weese and Bohs (2010) confirmed Lester’s hypothesis (e.g., Mace et al. 1999) that the cultivated eggplant has its closest relatives in Africa, although few Asian members of the Old World clade were examined. As part of of a larger monographic project on the African prickly solanums, examination of collections from South Africa identified a group of specimens distinct from the sympatric *Solanum campylacanthum*, *Solanum lichtensteinii*, and *Solanum linnaeanum* but with morphological similarity to the allopatric northern tropical African *Solanum cerasiferum* (Table 1). Preliminary molecular phylogenetic reconstruction using the nuclear ITS and *waxy* regions and the plastid *trnT-F* region confirms that this morphologically identified entity is distinct from *Solanum cerasiferum* and places it as sister to *Solanum linnaeanum* (S. Stern and L. Bohs, unpublished data). This new species is described here and the type selected from specimens in South African herbaria, following the recommendations of Smith and Figueiredo (2011).

## Taxonomic treatment

### Solanum umtuma

Voronts. & S.Knapp sp. nov.

urn:lsid:ipni.org:names:77116656-1

http://species-id.net/wiki/Solanum_umtuma

Figs 1 [Fig F2] –3 

#### Diagnosis.

Differs from *Solanum cerasiferum* Dunal by its cuneate to truncate leaf bases (versus short-attenuate leaf bases in *Solanum cerasiferum*), ovate foliaceous calyx lobes 7–10 mm long with between 30–80 prickles at anthesis on long-styled flowers (versus deltate to long-deltate membranous calyx lobes 4–7 mm long with only 0–20 prickles on long-styled flowers of *Solanum cerasiferum*); also differs from *Solanum linnaeanum* Hepper & P.-M.L.Jaeger by its shallow, obtuse to acute leaf lobes (versus deep, rounded leaf lobes in *Solanum linnaeanum*).

#### Type.

South Africa. Eastern Cape: Elliotdale District, The Haven [32°14'S, 28°54'E], forest margin, flower white, 17 Nov 1966, *J.L. Gordon-Gray 1017* (holotype: NU [NU-40255]).

#### Description.

Shrub, 0.5–1.5 m. Young stems erect, slender, moderately stellate-pubescent to glabrescent, with porrect sessile or variously stalked trichomes, the stalks to 0.2 mm long, the rays 6–8, ca. 0.2 mm long, the midpoints approximately the same length as the rays, armed with straight prickles 3–4 mm long, 1–2 mm wide at base, deltate, flattened, pale yellow-orange, glabrous, spaced 5–20 mm apart; bark of older stems glabrescent, green-brown to dark brown. Sympodial units plurifoliate. Leaves lobed; blades 8–20 cm long, 5–15 cm wide, 1.5–2 times longer than wide, elliptic, chartaceous, drying concolorous to weakly discolorous, green-brown, moderately stellate-pubescent on both surfaces, with porrect, sessile or stalked trichomes, the stalks to 0.2 mm long, the rays 6–8, 0.2–0.5(-0.8) mm long, the midpoints approximately the same length as the rays, with 5–20 prickles on both surfaces; the primary veins 4–6 pairs, the tertiary venation clearly visible abaxially and not visible adaxially; base cuneate to truncate; margins lobed, the lobes 3–4 on each side, 1–3 cm long, deltate, apically obtuse to acute, extending approximately 1/3 of the distance to the midvein, often with secondary lobing; apex obtuse to acute; petiole 1–3 cm long, approximately 1/6 of the leaf blade length, moderately stellate-pubescent, with 0–5 prickles. Inflorescences apparently lateral, 3.5–9 cm long, rarely branched, with 6–15(-20) flowers, 1–4 flowers open at any one time, weakly stellate-pubescent, with 0(-5) prickles; peduncle 1–3 mm long; pedicels 1–2.3 cm long in long-styled flowers, 0.8–1.2 cm long in short-styled flowers, erect to pendent, articulated at the base, moderately stellate-pubescent to glabrescent, with 0–20 prickles on long-styled flowers, unarmed on short-styled flowers; pedicel scars spaced 2–8 mm apart. Flowers 5-merous, heterostylous and the plants andromonoecious, with the lowermost 1–3 flowers long-styled and hermaphroditic, the distal flowers short-styled and functionally male. Calyx 11–22 mm long in long-styled flowers, 5–9 mm long in short-styled flowers, the lobes 7–10 mm long in long-styled flowers, 3–4 mm long in short-styled flowers, ovate and foliaceous in long-styled flowers, deltate in short-styled flowers, apically bluntly acute in long-styled flowers and acute to obtuse in short-styled flowers, moderately stellate-pubescent, with 30–80 prickles in long-styled flowers and 0–30 prickles in short-styled flowers. Corolla 2.5–3.3 cm in diameter in long-styled flowers, 1.5–2.5 cm in diameter in short-styled flowers, usually white or white with purple midveins, sometimes mauve, stellate, lobed for 1/4–1/2 of its length, the lobes ca. 7 mm long, ca. 10 mm wide in long-styled flowers, 6–10 mm long and 5–8 mm wide in short-styled flowers, broad-deltate, spreading, sparsely stellate-pubescent abaxially, the trichomes porrect, sessile or stalked, the stalks to 0.2 mm, the rays 5–8, 0.2–0.4 mm long, the midpoints approximately the same length as the rays. Stamens equal, with the filament tube 1–3 mm long, the free portion of the filaments ca. 0.5 mm long; anthers 5–6 mm long in long-styled flowers, 4.5–5.8 mm long in short-styled flowers, connivent, tapering, poricidal at the tips. Ovary glabrous, with a few stellate trichomes towards the apex; style 1.1–1.2 cm long in long-styled flowers, stout, straight or gently curved, moderately stellate-pubescent for most of its length. Fruit a spherical berry, 1(-2) per infructescence, 2.7–3.5 cm in diameter, the pericarp smooth, dark green with pale green and cream markings when young, yellow at maturity; fruiting pedicels 2–3 cm long, 1.2–2.2 mm in diameter at base, woody, pendulous, with 0–20 prickles; fruiting calyx not accrescent, covering 1/4–1/3 of the mature fruit, reflexed, with 10–80 prickles. Seeds ca. 100–200 per berry, 2.7–3.5 mm long, 2–2.5 mm wide, flattened-reniform, orange-brown.

**Figure 1.**
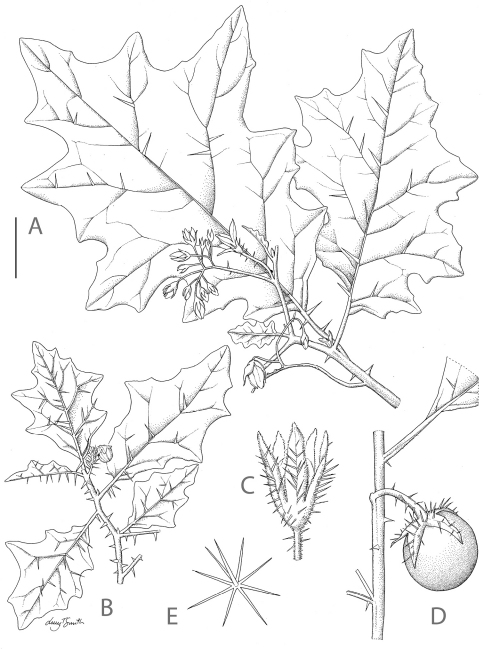
*Solanum umtuma*. **A** Habit with pronounced secondary leaf lobes and sparse prickles **B** Habit with few secondary leaf lobes and dense prickles **C** Calyx of a long-styled flower at anthesis **D** Fruiting branch **E** Porrect stellate trichome from the adaxial surface of a leaf. Scale bar: A, B, C = 3 cm; C = 1.5 cm; E = 0.5 mm. A, E from *Gerrard 295*; B-D from *Arnold 35934*. Drawn by Lucy T. Smith.

**Figure 2. F2:**
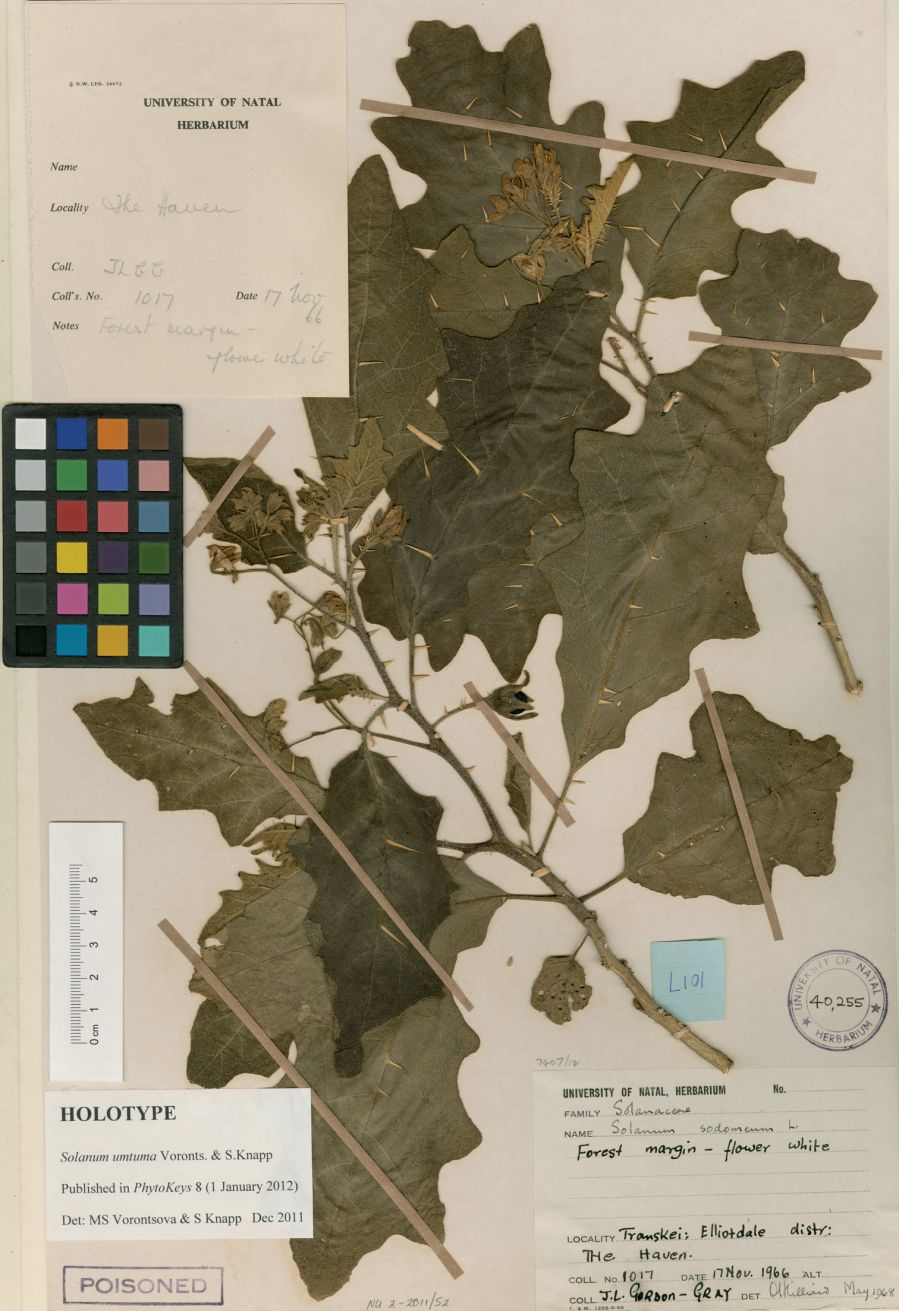
Photograph of the holotype of *Solanum umtuma* (*J.L. Gordon-Gray 1017*, NU-40255).

#### Distribution

**Figure 3.**
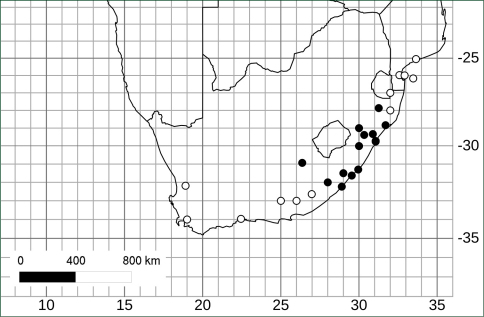
Distribution of *Solanum umtuma* (black circles) and its putative sister species *Solanum linnaeanum* (white circles) in southern Africa (specimen details for *Solanum linnaeanum* can be found on the Solanaceae Source website, http://www.solanaceaesource.org).

(Fig. 3). Endemic to South Africa in KwaZulu-Natal and Eastern Cape provinces (most specimens from KwaZulu-Natal); 50–1300 m elevation. *Solanum umtuma* is limited to the Maputaland-Pondoland Floristic Region (van Wyk and Smith 2001) and spans the Maputaland and Pondoland Centres of endemism.

#### Ecology.

Occasional on grassland, scrub, and forest edges, usually growing on sandy soil.

#### Etymology.

“Umthuma” is an isiXhosa vernacular name for many species of prickly *Solanum*; in the Xhosa language the “th” is pronounced as “t”, so we have here written the epithet phonetically as “*umtuma*”. The epithet is used here as a noun is apposition and thus not latinized to agree in gender.

#### Preliminary conservation status.

*Solanum umtuma* is a species of open and somewhat disturbed habitats (as are many prickly solanum species) and occupies an area of approximately 8000 km^2^ and appears to be relatively evenly distributed within that area (Fig. 3). Although not normally common where it occurs, it is not a species of immediate conservation concern.

#### Selected specimens examined.

South Africa. Eastern Cape: Transkei, outside Umtata [31°30'S, 29°00'E], 17 May 1975, *M.N.M. Arnold s.n.* (K [K000441994]); Port St Johns, 1 May 1899, *E.E. Galpin 2869* (K [K000545863]); Port St. Johns, 21 Dec 1932, *A.O.D. Mogg 1300* (K [K000545864]). —KwaZulu Natal: 50 km from Nongoma, 13 May 1975, *M.N.M. Arnold 35934* (K [K000795077]); Berea, 1862, *T. Cooper 1272* (BM [BM000887022], K [K000441992, K000441993]); Berea, 1862, *T. Cooper 1273* (K [K000441998, K000441999]); Noodsberg, Feb 2002, *T. Edwards 2973* (NU); location unknown, “Zululand”, received Jul 1865, *W.T. Gerrard 295* (BM [BM000887021], K [K000795076]); Umhlanga Rocks, 2 Sep 1966, *R.K. Grosvenor 168* (K [K000441995]); Weza forestry Area - beyond Lorna Doone [31°18'S, 29°57'E], 2 Jul 1986, *P.E. Hulley 134* (NU); Mkambati, Mkambati Enviromental Education Centre, 6 Apr 1988, *P.E. Hulley 230* (NU); Umgeni Park near Howick; Endulu Camp road, 18 Dec 1988, *P.E. Hulley & T. Olckers 279* (NU); 11 km N of Butterworth, 27 Apr 1990, *P.E. Hulley & T. Olckers* 333 (NU); Vernon Crookes Nature Reserve, 27 Apr 1990, *P.E. Hulley & T. Olckers 336* (NU); Umvoti, Umvoti valley S.W. of Mapumulo river bank, 9 Feb 1965, *E.J. Moll 1538* (K [K000442000]); Swart Umfolozi, Mpembeni, 1257 m, 27 Jan 2005, *L.S. Nevhutalu, LA. Nkuna, & E. van Wyk 921* (K [K000441997]); La Lucia, 14 Aug 1966, *R.G. Strey 6750* (K [K000441991]); Umhlanga Rocks, on gentle slopes above Umhlanga Rocks Hotel, 30 Dec 1959, *R.H. Watmough 461* (K [K000441996]); Ixopo, 22 Aug 1986, *J.O. Wirminghaus s.n*.(NU); Ngoye Forest, Zululand [28°50'S, 31°42'E], 17 Sep 1987, *J.O. Wirminghaus 628* (NU).

#### Discussion.

*Solanum umtuma* is a medium-sized subshrub with straight prickles, acute to obtuse leaf lobes, and large yellow fruits. It is almost certainly a close relative of the sympatric *Solanum linnaeanum*; the two species share long, leafy, prickly calyx lobes on long-styled flowers and fruits and differ primarily in the shape of their leaf lobes. *Solanum linnaeanum* is immediately recognisable by its quite deeply incised leaves with rounded lobes; a few intermediate specimens of *Solanum umtuma* have somewhat rounded lobes, e.g. *R.G. Strey 6750* (K000441991). Label data indicate that *Solanum umtuma* has white or only occasionally violet to mauve flowers, while *Solanum linnaeanum* always has purple flowers.

*Solanum umtuma* is morphologically very similar to *Solanum cerasiferum* and more superficially similar to other species with straight prickles and acute to obtuse leaf lobes, including the African highland *Solanum dasyphyllum* Schumach. & Thonn. (Solanaceae Source 2011) and *Solanum robustum* H.Wendl. of the New World (see Nee 1999). It is not sympatric with any of those species, so confusion is only possible in the herbarium. *Solanum umtuma* can be distinguished from *Solanum cerasiferum* by its cuneate to truncate leaf bases (versus short-attenuate leaf bases in *Solanum cerasiferum*), ovate foliaceous calyx lobes 7–10 mm long on long-styled flowers (versus deltate to long-deltate membranous calyx lobes 4–7 mm long on long-styled flowers in *Solanum cerasiferum*), and the densely spiny calyx of long-styled flowers with ca. 30–80 prickles at anthesis (versus flower calyces with only 0–20 prickles at anthesis in *Solanum cerasiferum*). *Solanum dasyphyllum* and *Solanum robustum* both have leaf blades that are markedly attenuate on the petiole and decurrent onto the stem, the stems are usually somewhat winged from these decurrent leaf bases. *Solanum umtuma* is sympatric with *Solanum lichtensteinii* and differs from it by its obtuse to acute leaf lobes (versus rounded leaf lobes in *Solanum lichtensteinii*).

Specimens of *Solanum umtuma* have sometimes been annotated as “*Solanum fuscatum* L.” or “*Solanum ferrugineum* Jacq.” These names are both widely misapplied. No original material of *Solanum fuscatum* L. has been located and the application of this name has been in doubt (Knapp and Jarvis 1990) and it has been proposed for rejection (Knapp 2011). *Solanum ferrugineum* Jacq. is the accepted name for a member of section *Torva* from the New World; this species occurs from Mexico to Costa Rica (Nee 1999; L. Bohs pers. comm.).

## Supplementary Material

XML Treatment for Solanum umtuma
